# Effectiveness of Subcutaneous Negative-Suction Drain on Surgical Site Infection After Ileostomy Reversal: A Propensity Score Matching Analysis

**DOI:** 10.3390/jcm14010236

**Published:** 2025-01-03

**Authors:** Ju Myung Song, Ji Hoon Kim, Moon Jin Kim, Chae Dong Lim, Yoon Suk Lee

**Affiliations:** 1 Department of Surgery, Incheon St. Mary’s Hospital, College of Medicine, The Catholic University of Korea, Incheon 21431, Republic of Korea; kjsong2002@naver.com (J.M.S.); ch1j8g@naver.com (M.J.K.); lcd90@cmcnu.or.kr (C.D.L.); 2 Department of Surgery, Seoul St. Mary’s Hospital, College of Medicine, The Catholic University of Korea, Seoul 06591, Republic of Korea; yslee@catholic.ac.kr

**Keywords:** wound drain, surgical site infection, ileostomy reversal

## Abstract

**Background/Objective:** Surgical site infection (SSI) is a leading common condition after ileostomy reversal (IR). However, evidence is unclear that subcutaneous negative-suction drainage (SND) reduces the incidence of SSI. This study aimed to investigate whether SND effectively reduced the incidence of SSI. **Methods:** We retrospectively analyzed the records of 531 patients who underwent IR at Incheon St. Mary’s Hospital between June 2005 and December 2020. SND was classified into two groups based on its presence or absence. The estimated risk of SSI was calculated using the surgical risk calculator of the American College of Surgeons National Surgical Quality Improvement Program (ACS NSQIP). After 1:1 propensity score matching (PSM) using the estimated risk of SSI, we analyzed the two group’s postoperative outcomes, including SSI rates. **Results:** After PSM, there was no difference in demographics between the two groups; however, the reversal interval was longer in the SND group than in the no SND group (193.3 ± 151.6 vs. 151.5 ± 141.0 days, *p* = 0.005). The incidence of SSI was lower in the SND group than in the no SND group (5.2% vs. 13.0%, *p* = 0.013). **Conclusions:** SND insertion can reduce the incidence of SSI during IR. Therefore, SND insertion should be considered as a basic technique for reducing SSI after IR.

## 1. Introduction

Surgical management plays an important role in the treatment of rectal cancer. Low anterior resections are performed on resectable rectal cancer. After performing low anterior resection for rectal cancer, the leak rate has been reported to be approximately 2–20% [1,2,3,4,5,6]. Anastomotic site leak is a severe complication of rectal cancer surgery that threatens not only quality of life but also life. Therefore, many patients with rectal cancer undergo a diverting ileostomy, and later, most undergo ileostomy reversal (IR).

In rectal cancer surgery, IR is often performed after an ileostomy creation, but various complications also occur. Surgical site infections (SSI) are the leading common complication of IR [7,8,9,10,11]. Several methods have been attempted to reduce SSI. Among them, subcutaneous negative-suction drainage (SND) is used to reduce the incidence of SSI. Several studies have been published on this topic [12,13,14,15,16,17,18,19]. However, the usefulness of SND remains unclear.

Additionally, there is considerable variation in the risk of SSI depending on patient characteristics and procedure type. Analysis of these factors is also one of the things to consider. The risk of SSI according to these factors can be predicted using the surgical risk calculator of The American College of Surgeons National Surgical Quality Improvement Program (ACS NSQIP) [20]. This calculator has proven its effectiveness and accuracy through many studies [21,22,23,24,25].

Therefore, this study aimed to analyze whether SND can reduce SSI incidence compared to the cases without SND. To analyze the role of SND, it is necessary to reduce bias to a minimum. To minimize the bias between the two groups, propensity score matching (PSM) was performed through the risk of SSI measurement using the surgical risk calculator of ACS NSQIP. Through this process, we analyzed the effectiveness of SND.

## 2. Methods

### 2.1. Study Design

**Figure 1:**
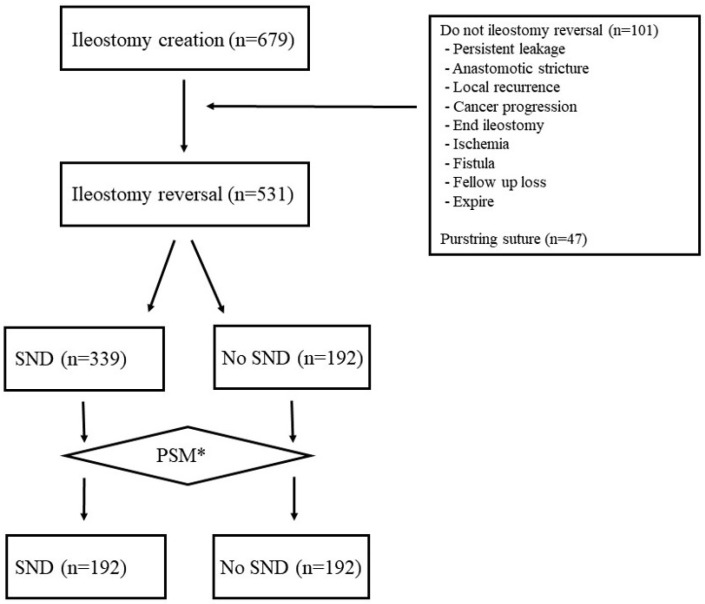
Patient selection flow diagram. * Propensity score matching.

This retrospective study included 679 patients who underwent rectal cancer surgery with diverting ileostomy between June 2005 and December 2020 (Figure 1). Of the 679 patients, 531 with primary wound closure were included in this study, excluding those who did not achieve IR, such as those with persistent leakage or anastomotic stricture, and those who underwent IR by other surgical methods, including purse-string suturing. Among them, 339 patients comprised the SND group, and 192 patients comprised the no SND group.

The estimated SSI risk was retrospectively calculated for all patients using the surgical risk calculator of the ACS NSQIP. Variables for risk calculation were age, sex, American Society of Anesthesiologists classification, steroid use for chronic conditions, ascites within 30 days preoperatively, systemic sepsis within 48 h preoperatively, ventilator dependence, disseminated cancer, diabetes, hypertension requiring medication, congestive heart failure within 30 days preoperatively, dyspnea, current smoking within 1 year, history of severe COPD, dialysis, acute renal failure, and body mass index.

To reduce selection bias, 1:1 PSM was performed using the estimated SSI risk and the presence of a parastomal hernia. Thus, the SND and no SND groups each comprised 192 patients.

Short-term morbidity included those that occurred within 1 month of the IR. In this study, SSI is defined as both superficial and deep incisional.

### 2.2. Surgical Procedure

**Figure 2:**
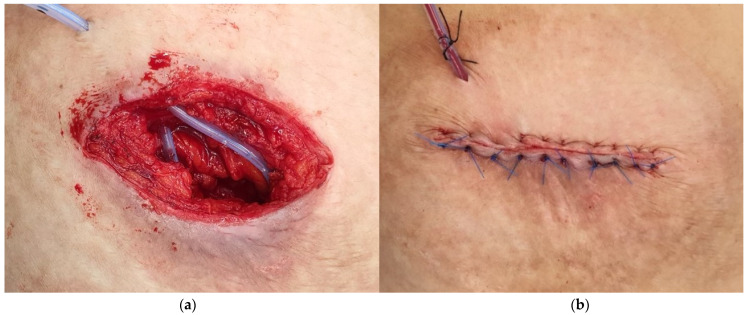
Subcutaneous negative-suction drainage (SND) insertion after fascia closure. (**a**) SND* insertion state. (**b**) Wound closure state. * Subcutaneous negative-suction drainage.

First-generation cephalosporins were administered before the skin incision. And we closed the openings of the ileostomy using non-absorbable sutures, and an elliptical skin incision was performed. The ileostomy was resected after separating it from the abdominal wall, and then functional end-to-end anastomosis was performed using linear staplers in all cases. After bowel anastomosis, we performed continuous suturing of the peritoneum using Vicryl #1-0. And then, the fascia and skin were closed interruptedly with absorbable (Vicryl #1-0) and non-absorbable (Nylon #3-0) sutures, respectively. Before skin suturing, the SND was placed on the fascia. A Jackson–Pratt drain was used for SND, and it was fixed to the skin at a location lateral to the skin incision. (Figure 2) Whether the drain would be used depended on the surgeon’s favor.

### 2.3. Postoperative Care

The drain was removed four days postoperatively. On postoperative day 4, the patient was discharged. However, if the amount of fluid from the drain was more than 20 mL for 24 h on the fourth postoperative day, the patient was discharged with the drain. After discharge, patients recorded the amount of drainage for 24 h without any additional dressing. Follow-up was conducted in the outpatient clinic on postoperative day 8, and it was confirmed whether the drainage volume of these patients had decreased to less than 20 mL or whether the drainage color was serous. So, the SND was removed on postoperative day 8 at the outpatient clinic. The skin suture material was removed between postoperative days 7 and 14.

### 2.4. Statistics

R software version 4.0.0 (Foundation for Statistical Computing, Vienna, Austria) was used as the statistical tool. An independent *t*-test, chi-squared test, and Fisher’s exact test were used for statistical analysis. Statistical significance was set at *p* < 0.05. PSM is used to reduce bias in retrospective studies in which a randomized control study is unable to be conducted. We, therefore, employed PSM. Propensity scores were calculated using bivariate logistic regression. A 1:1 PSM was performed for the SND and no SND groups. The variables considered for matching were SSI risk estimated using the ACS NSQIP risk calculator and the presence of a parastomal hernia.

After PSM, statistical analysis was performed to compare the two groups using the same method. The primary and secondary outcomes were the surgical site infection rate and morbidity, respectively.

The study protocol was reviewed and approved by the Institutional Review Board of Incheon St. Mary’s Hospital (IRB No. OC2IRISI0094). The requirement for informed consent was waived.

### 2.5. Research Ethics

The study protocol was reviewed and approved by the Institutional Review Board of Incheon St. Mary’s Hospital (IRB No. OC2IRISI0094). Prior consent requirements have been waived. The approval date is 20 August 2021.

## 3. Results

Our retrospective study included 531 patients who underwent IR between June 2005 and December 2020: 339 comprised the SND group, and 192 comprised the no SND group. First, we analyzed patient characteristics between the two groups. Table 1 shows patient characteristics between the SND and the no SND groups. Before PSM, the patients in the SND group were older than those in the no SND group (63.1 ± 12.5 vs. 60.6 ± 11.0, *p* = 0.023). However, there were no significant differences in other variables. To reduce bias, PSM was implemented, and statistical analysis was performed. After PSM, the age was similar between the two groups (60.8 ± 11.9 vs. 60.6 ± 11.0, *p* = 0.859), and there were no significant differences in demographics, excluding the reversal interval (193.3 ± 151.6 vs. 151.5 ± 141.0 days, *p* = 0.005). There were no significant differences between the two groups in other variables, including estimated SSI risk by the ACS NSQIP risk calculator (5.5 ± 1.1 vs. 5.6 ± 1.1%, *p* = 0.870), diagnosis (*p* = 0.659), and rate of parastomal hernia (8.3% vs. 8.3%, *p* = 1.000). There were no differences in cosmetic results and patient satisfaction between the two groups.

Table 2 is a comparative analysis of postoperative outcomes between the SND and the no SND groups. Before PSM, there was a significant difference in short-term morbidity between the two groups (5.6% vs. 14.1%, *p* = 0.002). Among short-term outcomes, SSI had a significantly lower incidence in the SND group compared to the no SND group (3.5% vs. 13.0%, *p* < 0.001). The incidence of incisional hernia was also significantly lower in the SND group (0.9% vs. 4.2%, *p* = 0.025). Also, to minimize bias, PSM was performed, and the analysis was performed again. After PSM, there was no difference between the SND group and the no SND group in short-term morbidity. However, the incidence of SSI was significantly lower in the SND group (5.2% vs. 13.0%, *p* = 0.013). There was no significant difference in the incidence of incisional hernia between the two groups (1.6% vs. 3.6%, *p* = 0.336).

## 4. Discussion

Anastomotic site leakage is a serious complication in rectal cancer surgery. This complication is a major obstacle for surgeons treating rectal cancer and causes difficulties in further treatment. To overcome this situation, ileostomy creation is performed. Afterward, the surgeon confirms that the anastomotic site is intact by proctoscopy and performs IR. This allows patient treatment to proceed without anastomotic site leakage [26].

IR is considered to be a contaminated surgery. Previously, the incidence of SSI after IR with primary wound closure without a drain was reported to be approximately 18% [7,8,9,10,11]. Despite the high incidence of SSI reported in many studies, many surgeons tend to maintain existing methods rather than making efforts to correct them [27]. Therefore, considerable efforts are required to reduce the risk of SSI during IR.

We believed that the most effective method to reduce SSI was to use SND insertion on the subcutaneous layer. So, we performed wound closure using SND insertion.

To reduce the SSI rate, there are several skin closure methods for IR other than primary skin closure without a drain. Other methods include delayed wound closure, purse-string suture closure, negative-pressure wound therapy (NPWT), and primary closure with a negative suction drain. However, the optimal method of skin closure to reduce the SSI rate after IR remains debatable.

Delayed wound closure has been reported to lower the SSI rates in contaminated wounds. However, SSI rates after IR have shown contradictory results [8,10,28,29]. Additionally, delayed wound closure requires a longer healing period than primary wound closure.

The SSI rate in purse-string sutures is lower than that in primary wound closures [8,30]. Because of this relatively low SSI rate, the American College of Surgeons recommends closing wounds after IR using the purse-string technique [31]. And the Italian Society of ColoRectal Surgery (SICCR) reported that surgeons with more experience in IR tend to prefer purse-string sutures in wound approximation [32]. However, this method requires approximately one month for complete wound healing and frequent wound dressings because of exudates. Because of these difficulties, patient satisfaction and economic effectiveness is also low.

Recently, studies on the application of NPWT after IR have been published. NPWT has been reported to result in lower SSI rates than those without NPWT, with no delay in wound healing [33,34,35]. However, NPWT requires special equipment and incurs additional costs. Moreover, NPWT is not covered by health insurance in South Korea.

The results of previous studies on primary closure using suction drains are contradictory. Lauscher et al. reported similar SSI rates between SND and no SND groups (14% vs. 17%, *p* = 0.68). The drain was removed on postoperative day 2, regardless of the amount of fluid [14]. However, another study reported lower SSI rates in the SND group than in the non-SND group (1.2% vs. 12.5%, *p* = 0.001). The drain was removed on postoperative day 3 [13]. In this study, we removed the drain between postoperative days 4 and 7. We believe that the early removal of drains, regardless of the drainage volume, may not help prevent SSI. Therefore, additional studies are required to determine the appropriate timing for drain removal.

We believe that the reason for the low SSI risk in the SND group was that correctable infection-causing factors, such as seroma or hematoma, can be efficiently removed from the surgical site by placing a closed suction drain. We believe that negative pressure increases blood flow, which promotes wound healing.

A parastomal hernia may be a risk factor for SSI because of its larger dead space [36,37]. However, to the best of our knowledge, no studies have been conducted on this topic. Therefore, we considered the presence of a parastomal hernia as a variable in the propensity score.

Overall, we found that SND insertion had a preventive effect in reducing the incidence of SSI in IR. Therefore, SND insertion should be considered as a basic technique for reducing SSI after IR.

## 5. Limitations and Strengths

The study limitations include its single-center retrospective design and the fact that the decision to insert an SND was based on the surgeon’s preference.

However, to overcome these limitations, we attempted to remove bias using the SSI risk calculated by the ACS NSQIP calculator as a variable for PSM beyond simply analyzing PSM. This is because the SSI risk calculated using the ACS NSQIP calculator is a weighted result for each risk factor. Moreover, we considered parastomal hernia, which has not been considered by any other researcher, as a risk factor for SSI.

## 6. Conclusions and Future Perspectives

SND insertion can reduce the incidence of SSI during IR. Therefore, SND insertion should be considered as a basic technique for reducing SSI after IR. Eventually, RCT-controlled trials are needed to clarify the conclusions of our study.

## Figures and Tables

**Table 1:** Patient characteristics before and after propensity score matching.

	Before Propensity Score Matching			After Propensity Score Matching		
	SND (n = 339)	No SND (n = 192)	*p*-Value	SND (n = 192)	No SND (n = 192)	*p*-Value
Sex			0.815			0.317
Male	223 (65.8%)	129 (67.2%)		139 (72.4%)	129 (67.2%)	
Female	116 (34.2%)	63 (32.8%)		53 (27.6%)	63 (32.8%)	
Age (years)	63.1 ± 12.5	60.6 ± 11.0	0.023	60.8 ± 11.9	60.6 ± 11.0	0.859
BMI * (kg/m^2^)	23.8 ± 3.1	24.0 ± 3.4	0.454	23.8 ± 3.0	24.0 ± 3.4	0.434
ASA classification +			1.000			0.638
1 and 2	324 (95.6%)	184 (95.8%)		181 (94.3%)	184 (95.8%)	
3	15 (4.4%)	8 (4.2%)		11 (5.7%)	8 (4.2%)	
Estimated SSI risk by ACS NSQIP § risk calculator (%)	5.4 ± 1.1	5.6 ± 1.1	0.224	5.5 ± 1.1	5.6 ± 1.1	0.870
Diagnosis			0.851			0.659
T-colon ca. €	1 (0.3%)	0 (0.0%)		1 (0.5%)	0 (0.0%)	
S-colon ca. ¥	14 (4.1%)	8 (4.2%)		10 (5.2%)	8 (4.2%)	
RS colon ca. $	1 (0.3%)	0 (0.0%)		0 (0.0%)	0 (0.0%)	
Rectal ca. £	320 (94.4%)	183 (95.3%)		179 (93.2%)	183 (95.3%)	
FAP ♀	3 (0.9%)	1 (0.5%)		2 (1.0%)	1 (0.5%)	
Reversal interval (days)	168.0 ± 128.9	151.5 ± 141.0	0.173	193.3 ± 151.6	151.5 ± 141.0	0.005
Parastomal hernia	49 (14.5%)	16 (8.3%)	0.054	16 (8.3%)	16 (8.3%)	1.000

* Body Mass Index, + American Society of Anesthesiologists Classification, § American College of Surgeons National Surgical Quality Improvement Program, € Transvers colon cancer, ¥ Sigmoid colon cancer, $ Rectosigmoid colon cancer, £ Rectal cancer, ♀ Familial adenomatous polyposis.

**Table 2:** Postoperative outcome before and after propensity score matching.

	Before Propensity Score Matching			After Propensity Score Matching		
	SND (n = 339)	No SND (n = 192)	*p*-Value	SND (n = 192)	No SND (n = 192)	*p*-Value
Short-term Morbidity	19 (5.6%)	27 (14.1%)	0.002	17 (8.9%)	27 (14.1%)	0.199
SSI *	12 (3.5%)	25 (13.0%)	<0.001	10 (5.2%)	25 (13.0%)	0.013
Ileus	6 (1.8%)	4 (2.1%)	0.320	6 (3.1%)	4 (2.1%)	0.254
Etc.	2 (0.6%)	1 (0.5%)	0.752	2 (1.0%)	1 (0.5%)	0.701
Incisional hernia	3 (0.9%)	8 (4.2%)	0.025	3 (1.6%)	7 (3.6%)	0.336

* Surgical site infection.

## Data Availability

Data are contained within the article.
